# The effects of ultrasonic processing on the antioxidant activity of *Geotrichum candidum* LG-8 and its cell wall extracts

**DOI:** 10.3389/fnut.2024.1334956

**Published:** 2024-02-21

**Authors:** Fengping Jiao, Xianping Cui, Xiaodi Gong, Guozheng Jiang, Jinbiao Wang, Ling Meng

**Affiliations:** ^1^School of Public Health, Shandong First Medical University and Shandong Academy of Medical Sciences, Jinan, Shandong, China; ^2^Division of Hepatobiliary and Pancreatic Surgery, Affiliated Provincial Hospital, Shandong First Medical University and Shandong Academy of Medical Sciences, Jinan, Shandong, China; ^3^Yantai Hengyuan Bioengineering Co. Ltd., Yantai, Shandong, China

**Keywords:** *Geotrichum*, ABTS, antioxidant, nanopolysaccharide, ultrasonic processing

## Abstract

**Introduction:**

Extraction techniques that influence cell wall polysaccharides (EPS) is crucial for maximizing their bioactivity. This study evaluates ultrasound technology for extracting antioxidant polysaccharides from *Geotrichum candidum* LG-8, assessing its impacton antioxidant activity.

**Methods:**

Ultrasound extraction of EPS from *G. candidum* LG-8 was optimized (18 min, pH 7.0, 40 W/cm^2^, 0.75 M NaCl). ABTS scavenging efficiency and monosaccharide composition of LG-EPS1 and LG-EPS3 were analyzed using Fourier-transform infrared spectroscopy (FTIR) and scanning electron microscopy (SEM).

**Results:**

The Results showed that ultrasonic treatment markedly increased the ABTS radical scavenging efficiency of LG-8 cells by 47%. At a concentration of 1 mg/mL, the ultrasonically extracted LG-EPS1 and LG-EPS3 polysaccharides exhibited significant ABTS radical scavenging efficiencies of 26% and 51%, respectively. Monosaccharide composition analysis identified mannose and glucose in LG-EPS1, while LG-EPS3 was primarily composed of mannose. FTIR spectra verified the polysaccharides' presence, and SEM provided visual confirmation of the nanoparticle structures characteristic of LG-EPS1 and LG-EPS3.

**Discussion:**

This research not only underscores the technological merits of ultrasound in polysaccharide extraction but also highlights the potential of *G. candidum* LG-8 derived polysaccharides as valuable bioactive compounds for antioxidant utilization.

## 1 Introduction

The fungi used in the investigation belong to *Geotrichum*, a yeast-like strain isolated from Kefir milk in Tibet, China. *Geotrichum* cells are rich in proteins and polysaccharides, and therefore, these fungi are candidates for food and fodder. Wang et al. (1) used *G. candidum* and *Lactobacillus plantarum* to make a fermentative beverage. Ferreira et al. (2) immobilized lipase from *Geotrichum candidum* to obtain a biocatalyst. It not only has a low requirement for nutrients but also strong adaptability and fast growth, indicating that the fungus is applicable in industrialization.

The yeast cell wall is generally composed of polysaccharides, which are associated with mannose and glucose. The polysaccharides released from the cells can be divided into two types: water-soluble and water-insoluble (3, 4). However, they are also known for connecting with proteins by covalent bonding (2). Therefore, it is valuable to consider the presence of proteins when extracting polysaccharides as glycoproteins always have better functions than polysaccharides. Polysaccharides are macromolecular polymers with a variety of excellent functions, such as antioxidant and anti-tumor functions. Therefore, they are widely used in food, medicine, and cosmetics (5–7). The cell walls of yeast also have variable functional groups that show strong activities in the immune system (3). Hudson et al. (8) reported that the outer layer of the *Saccharomyces boulardii* cell wall containing mannose plays an important role in mucosal immune system health. Liu and Huang showed that polysaccharides extracted from yeast cell walls had an antioxidative function (4). Therefore, antioxidant activity is regularly used as an indicator for evaluating the functions or releasing efficiencies of extracts in cell walls.

Modern techniques used for the extraction and purification of polysaccharides and proteins generally include acid, alkali, and ultrasonic methods (9–11). However, polysaccharides and proteins have variable functions, possibly due to their purity, proportions, molecular weight, and spatial structure, which are influenced by extraction methods (12). Shang et al. (9) showed that monosaccharide compositions of *Medicago sativa* polysaccharides extracted by four different methods were the same but the proportions were different. Therefore, the extraction method possibly influences the antioxidant activities of *Geotrichum candidum* LG-8 polysaccharides.

In this study, ultrasonic investigations of the antioxidant activity of *Geotrichum candidum* LG-8 and extracting efficiency of its cell wall components were undertaken. The influences of several factors on the extraction of polysaccharides and proteins from the *Geotrichum candidum* LG-8 cell wall using this method were evaluated. Additionally, the antioxidant activities of cells, ultrasonic-treated cells, and cell wall polysaccharides (EPS) were studied. The monosaccharide components of the extracted water-soluble polysaccharides were analyzed. An optimized ultrasonic process is expected to help provide a potential resource for antioxidants.

## 2 Materials and methods

### 2.1 Strain and culture

*Geotrichum candidum* LG-8 (MK640636) was provided by the Food Microbiology Laboratory of Nanjing Agricultural University (Nanjing, China). It was isolated from kefir milk, a traditional dairy product from Tibet, China. The fungus was activated in YPD agar containing 20 g/L of peptone, 20 g/L of fructose, and 10 g/L of yeast extract and cultivated three times in YPD broth at 30 °C ± 2°C and 180 rpm for 24 h. LG-8 cells were then collected by centrifugation at 3000 rpm and 4°C for 10 min. After being washed in PBS three times, the cells were stored at 4°C in PBS.

### 2.2 Ultrasound treatment

The ultrasound process was optimized through single-factor tests with extraction efficiency as the index. Note that the extraction efficiency refers to the ratio of protein or polysaccharide mass released to total yeast mass. The typical parameters of ultrasound extraction were set as follows: LG-8 cells at 0.05 g, an NaCl concentration of 1 M (pH7), an ultrasonic intensity of 40 W/cm^2^, an ultrasonic temperature of 4°C, and an ultrasonic time of 18 min. The specific treatment was processed as follows:

First, *Geotrichum candidum* LG-8 cells were dispersed in NaCl solution and maintained at 4°C. Sonication was conducted using an ultrasonic generator (XO-1000D, Xianou, China) with a probe (No. 6) in continuous mode. This No 0.6 probe was inserted into the liquid surface for 1 cm. Second, the effects of LG-8 biomass (0.25 g/L, 0.5 g/L, 1.0 g/L, 1.5 g/L, 2.0 g/L, 2.5 g/L, 3.0 g/L, and 3.5 g/L), ultrasonic intensity (10 W/cm^2^, 20 W/cm^2^, 30 W/cm^2^, 40 W/cm^2^, 50 W/cm^2^, and 60 W/cm^2^), NaCl concentrations (0.25 M, 0.75 M, 1 M, 1.25 M, 1.5 M, and 1.75 M), ultrasonic time (3 min, 6 min, 9 min, 12 min, 15 min, 18 min, 21 min, 24 min, 27 min, 30 min, and 36 min), and pH (2, 3, 4, 5, 6, 7, 8, 9, and 10) on extracting polysaccharides and proteins from the cell walls of *Geotrichum candidum* LG-8 were investigated. Third, after each ultrasound treatment, a 0.5 mL suspension was collected immediately by centrifugation at 940 g for 30 min, diluted with 4.5 mL of water to measure the amount of protein and polysaccharide released. Extraction efficiency was calculated using the following Equation (1):


(1) $$\begin{array}{l} Extraction\;efficiency\;\left(\%\right)=\left[\frac{A_{extracts}}{A_{LG-8}}\right]\times 100 \end{array}$$

A_LG−8_ is the total yeast (*Geotrichum candidum* LG-8) cell mass in each above ultrasound treatment. A_extracts_ is the protein or polysaccharide mass measured according to the methods in Section 2.3.

### 2.3 Analysis of the polysaccharides and proteins released

Diluted ultrasound supernatant obtained in Section 2.2 was used to measure the amount of protein and polysaccharide released from the cell wall. For the analysis of polysaccharides, glucose was taken as the standard. The concentration of polysaccharide in ultrasonic suspension (Section 2.2) was measured according to a modified phenol-sulfuric acid method (13). Briefly, 0.5 mL of suspension was blended through the addition of phenol (0.6%, 0.5 mL) and sulfuric acid (2.5 mL) for 20 min. After being cooled within 5 min to room temperature, the absorbance at 490 nm was determined (UV752, Spsic, China) so that the amount of polysaccharide could be calculated. The linear range of this method was 5–100 mg glucose/L, resulting in the equation Y=11.918x+0.0318 (R^2^ = 0.9976). The polysaccharide mass released was calculated according to the following Equation (2):


(2) $$\begin{array}{l} Polysaccharide\;mass\;=\left[\frac{Y-0.0318}{11.918}\right]\times V\times Z \end{array}$$

where Y is the absorbance of the extracted polysaccharide in the ultrasound supernatant, V is the total volume of the ultrasound supernatant, and Z is the dilution multiple of the ultrasound supernatant.

For the analysis of protein, bovine serum albumin (BSA) was used as the control group. The concentration of BSA or released protein was measured according to the Coomassie brilliant blue (CBB) method. A volume of 1 mL of sample was added to 5 mL of CBB and incubated for 5 min at room temperature. The absorbance at 595 nm was determined (UV752, SPSIC, China) so that the amount of protein could be calculated. The linear range of this method was 5 mg BSA/L−100 mg BSA/L, resulting in the equation Y = 6.4757x + 0.0397 (R^2^ = 0.997856). The protein mass released was calculated according to the following Equation (3):


(3) $$\begin{array}{l} Protein\;mass\;=\left[\frac{Y-0.0397}{36.4757}\right]\times V\times Z \end{array}$$

where Y is the absorbance of the extracted protein in ultrasound supernatant, V is the total volume of the ultrasound supernatant, and Z is the dilution multiple of the ultrasound supernatant.

### 2.4 Extraction and purification

The suspension was obtained under the ultrasonic conditions optimized in Section 2.2. Then, it was dialyzed in a dialysis bag (MWCO: 8000 Da−14000 Da) to remove small molecules and salts. The solution was named LG-EPS. After being concentrated and freeze-dried, the crude cell-wall extracts obtained were named LG-EPS1.

Purification of the crude extract LG-EPS1 was carried out in two steps. First, trichloroacetic acid solution (TCA, 4%) was added to LG-EPS1 to a concentration of 8%. After storage at 4°C overnight, the precipitated protein was removed by centrifuging at 940 g for 30 min. The EPS was then added in ethanol (3:1, v/v), stored at 4°C for 24 h, and then centrifuged again. After being washed with ethanol, the sediment was dissolved in deionized water and decanted into dialysis bags for deionization. During the 3-day dialysis at 4°C, the deionized water was changed every 4 h. The cell wall polysaccharide (EPS) was ultimately dried in a lyophilizer (Heto PowerDry LL3000, Thermo, USA) and was named LG-EPS2.

Afterwards, 5 mg of the freeze-dried LG-EPS2 undergoing preliminary purification was dissolved in 2 mL of deionized water and then purified by gel chromatography with a Sephadex G-100 gel column (1.6 × 60 cm). The eluent was deionized water. The elution rate was 1 mL/min. An aliquot of 10 mL of elution solution per tube was collected using an automatic collection device (BS-100A, Huxi, China). The concentration of polysaccharides was determined according to the method shown in Section 2.4. The eluents containing the same components were merged into one tube. Different polysaccharide components were obtained by concentrating with decompression and freeze-drying. In the present purification, only one component was obtained, which we marked LG-EPS3.

### 2.5 Analysis of ABTS [2,2'-azinobis-(3-ethylbenzthiazoline-6-sulphonate)] radical scavenging activity

ABTS [2,2'-azinobis-(3-ethylbenzthiazoline-6-sulphonate)] radical scavenging activity of extracts and cells was analyzed according to the methods described by Rumpf et al. (14). Briefly, an ABTS solution was prepared by mixing 2.45 mM of aqueous K_2_S_2_O_8_ and 7 mM aqueous ABTS (1:1, v/v) at 25°C in the dark for 24 h. Afterwards, 0.700 at 734 nm was achieved by diluting the stock solution with 50% (v/v) ethanol. A volume of 40 μL of sample (0.1 mg/mL) was directly used to evaluate its total antioxidant activity by adding 160 μL of ABTS and K_2_S_2_O_8_ (1: 2, v/v). The mixture was incubated at 37°C for 6 min and subsequently centrifuged at 940 g for 10 min. The OD values were detected at 734 nm using an ultraviolet spectrophotometer (UV752, SPSIC, China). Distilled water was used instead of the sample as a control. ABTS radical cation scavenging efficiency was calculated according to the following Equation (4):


(4) $$\begin{array}{l} ABTS\;radical\;cation\;scavenging\;efficiency\;\left(\%\right)\\ =\left[\frac{A_{control}-A_{sample}}{A_{control}}\right]\times 100 \end{array}$$

where A_control_ is the absorbance of the control group under identical conditions and A_sample_ is the absorbance of the sample group under identical conditions (LG-EPS1, LG-EPS3, and *G. candidum* LG-8 cells before or after ultrasonic processing).

### 2.6 Determination of monosaccharide composition

The monosaccharide composition of crude LG-EPS1 or column-purified LG-EPS3 was determined by pre-column high-performance liquid chromatography (HPLC). Specifically, 5 mg of LG-EPS1 or LG-EPS3 was firstly acidified at 120°C for 2 h through the addition of 2 mL of trifluoroacetic acid (TFA, 2 M). The acidulated products (monosaccharide) were then dried at 50°C under negative pressure. Residual TFA was removed in the presence of methanol (5 mL) by a rotary evaporator; this process was repeated at least three times.

The monosaccharide was dissolved in 0.5 mL of deionized water for later derivatization. A volume of 100 μL of NaOH (0.6 M) and 100 μL of PMP (1-phenyl-3-methyl-5-pyrazolone) methanol solution (0.5 M) were added in turn to 100 μL of the monosaccharide. The mixture reacted at 70°C for 100 min. After cooling to 24°C, the mixture was neutralized by 50 μL of HCl (0.3 M) and dried at 50°C under negative pressure. Distilled water (1 ml) and 1 mL of chloroform were added to the dried mixture. The chloroform layer was carefully discarded. After repeating the extraction procedure three times, aqueous solution was filtrated using a filter membrane (0.45 μm). The monosaccharide composition was analyzed by HPLC (1100, Agilent, USA) using an ultraviolet detector (245 nm) and a PR-C18 column (4.6 mm × 250 mm, 5 μm, Venusil, USA) maintained at 30°C. Furthermore, the sample was eluted at a flow rate of 1 mL/min with phosphate-buffered saline (PBS, 0.05 M, pH 6.8) and acetonitrile (82: 18, v/v). The monosaccharide references (glucose, fructose, mannose, rhamnose, arabinose, and galactose) were also pretreated and determined in the same conditions. The molar ratio of sample monosaccharides was calculated depending on the peak area of those monosaccharides.

### 2.7 SEM and TEM analysis

For the SEM analysis, the morphology of LG-EPS1 and LG-EPS3 was visualized using SEM (S-3000N, Hitachi, Japan). The samples were pretreated and visualized at magnification according to methods reported by Meng et al. (15). Briefly, a critical point dryer with CO_2_ was used to dry the sample. After being placed on coverslips and sputtered with gold particles, samples were visualized by SEM (S-3000N, Hitachi, Japan).

For the TEM analysis, the morphology of *Geotrichum candidum* LG-8 was visualized using TEM (S-3000N, Hitachi, Japan) according to methods described by Li et al. (16). Specifically, cells were sequentially fixed with glutaraldehyde (2.5%, v/v) and 0.5 mL of osmium tetroxide (1%) for 12 h and 2 h at 4°C, respectively. After removing excess fixatives using sodium phosphate buffer (0.1 M, pH 7.4), cells were treated with ethanol solutions (70%, 80%, 90%, and 100%), propylene oxide (15 min), a mixture of propylene oxide (Epon, 1:1, v/v, 1h), propylene oxide (Epon, 1:3, v/v, 2h), and pure Epon in order. Finally, resin blocks were cut into ultrathin sections (65 nm), which were stained with uranyl acetate and lead citrate. The sections were mounted on copper grids and observed using a TEM (JEM2100, JEOL, Japan).

### 2.8 FTIR analysis

Lyophilized LG-EPS1 and LG-EPS3 were analyzed using an FTIR analyzer (Tensor-27, Bruker, Germany). The sample (1 mg) and 100 mg of KBr powders were mixed and detected in a broad wavenumber range (4000 cm^−1^-400 cm^−1^).

### 2.9 Statistical analysis

All the samples were prepared in triplicate. The data were analyzed using one-way ANOVA using Statistical Package for the Social Sciences (SPSS) software version 20.

## 3 Results and discussion

### 3.1 Factors affecting EPS production

The cells were treated by the interaction with cavitation bubbles. Logically, the number of cavitation bubbles and the qualities of the cell wall can influence extraction efficiency. Therefore, it is worth evaluating the effects of processing parameters on extracting EPS from the cell wall of *G. candidum* LG-8. On the other hand, polysaccharides in this yeast are the main components of the cell wall, while most of the proteins are intracellularly segregated by the cell membrane. We thereby assessed the extent of the damage to the cell wall and membrane by polysaccharide and protein content, respectively.

#### 3.1.1 The influence of biomass

**Figure 1:**
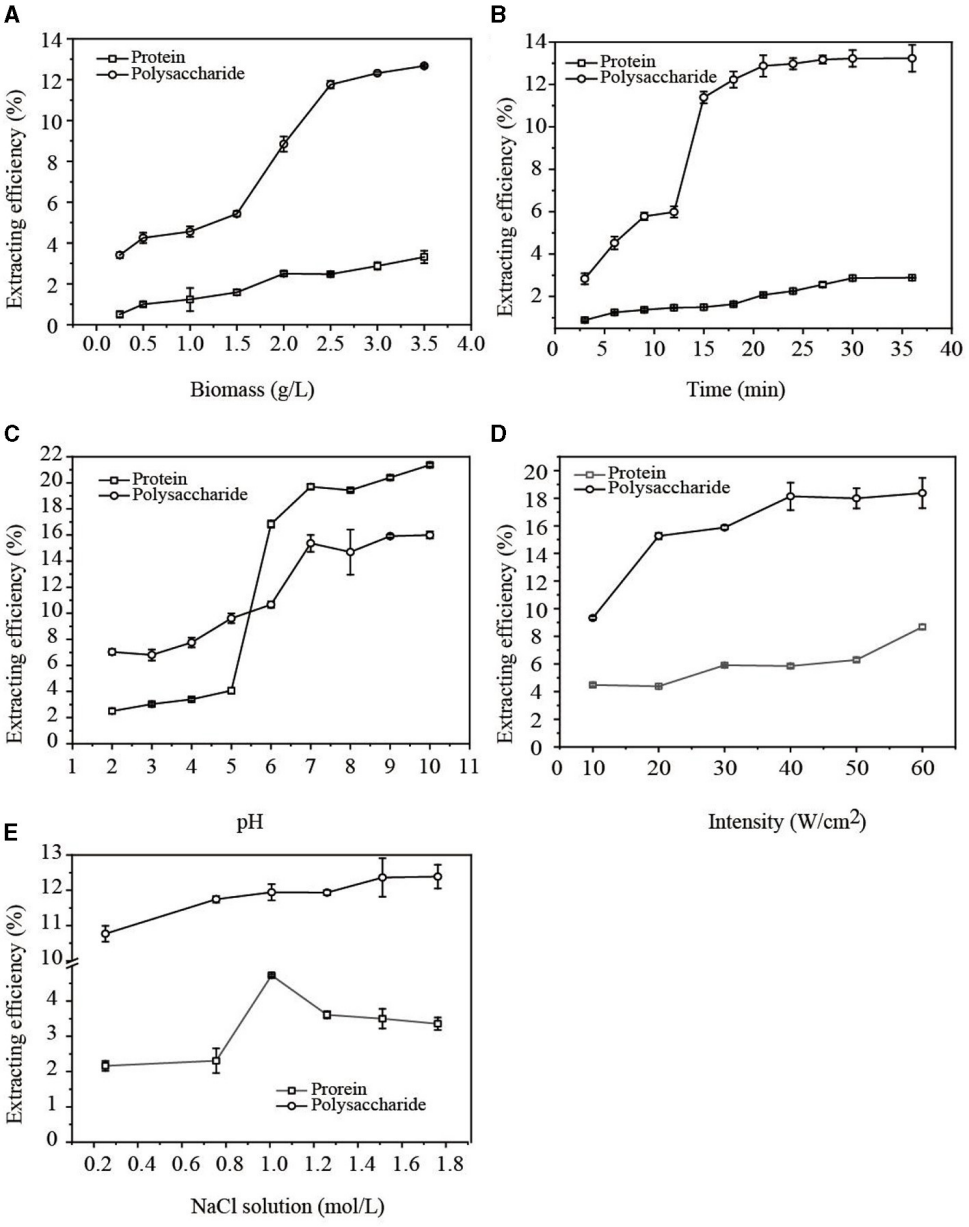
Factors [**(A)** biomass; **(B)** time; **(C)** pH; **(D)** intensity; **(E)** NaCl concentration] affecting the extraction efficiency of EPS.

The influence of *G. candidum* LG-8 biomass on extracting water-soluble polysaccharides and proteins in cell walls using the ultrasonic method was studied. In Figure 1A, when *G. candidum* LG-8 biomass increased from 1.5 g/L to 2.5 g/L, the extraction efficiency of polysaccharides and proteins increased from 5.34% and 1.58% to 11.76% and 2.48%, respectively. As *G. candidum* LG-8 biomass was more than 2.5 g/L, the extraction rate of polysaccharides slowly increased while that of proteins tended to be stable. The results indicated that increasing biomass was conducive to improving the extraction efficiency of polysaccharides but did not significantly increase the extraction rate of proteins. By increasing *G. candidum* LG-8 biomass, the contact density of the cells, ultrasonic cavitation bubble, and source of polysaccharides increased. However, the increased density is not favorable for generating cavitation bubbles, which also leads to the dispersion of interaction between cavitation bubbles and *G. candidum* LG-8 cells. Therefore, the maximum biomass (3.5 g/L) was applied as one of the conditions for extracting polysaccharides in the study.

#### 3.1.2 The influence of time

The influence of ultrasonic time on polysaccharide and protein release within 36 min was studied. In Figure 1B, the results showed that the extraction efficiencies of the polysaccharide and protein increased from 2.84% and 0.89% with time, reaching maximums of 13.23% and 2.89% at 21 and 30 min, respectively. Indeed, some investigations have also documented a similar phenomenon, observing a rise-equilibrium curve in the release of polysaccharides and proteins over time (17). The findings suggested that cavitation firstly attacked the cell wall, which can be responsible for the early equilibrium of releasing polysaccharides, and later, the saturation of releasing protein. On the other hand, previous reports showed that the released polysaccharides increasing with time may be due to the increasing soluble content of poorly water-soluble polysaccharides (18). The second factor also could be considered to be responsible for a rapid increase in the extraction efficiency (5.98%) of polysaccharides at 12 min (Figure 1B). Compared with the previous studies, the released contents of soluble, poorly soluble, and insoluble polysaccharides in *G. candidum* cell walls are more stable. When the polysaccharides of the cell wall were extracted to the maximum extent, the extraction efficiency of polysaccharides reached the equilibrium. However, the extraction efficiencies of polysaccharides and proteins at 18 min were 12.23% and 1.64%, respectively, which increased only ~1% at the equilibrium time. In the above context, 18 min was applied as an optimum condition for extracting polysaccharides in the study.

#### 3.1.3 The influence of pH

The solution pH is an important factor that could influence the extraction efficiency of natural products. Chen et al. (11) reported that the optimal condition for extracting PPS by ultrasound was pH 7. Azat et al. (19) applied ultrasound to extract polysaccharides from Ulva rigida at the optimal pH of 4.92. To maximize the extraction efficiency of polysaccharides or proteins, pH was therefore optimized in our study. Figure 1C shows that the extraction efficiencies of polysaccharides and proteins increased with rising pH (from 2 to 7). In alkaline solutions with a pH higher than 7, the extraction rate of polysaccharides tended to be stable, but the extraction rate of proteins continued to increase. This result showed that the extraction efficiency of protein was more dependent on pH.

Alkaline extraction and acid precipitation were frequently used to extract protein. A weak acidic environment, especially one with a pH of 6.0–7.0, is favorable for the extraction of polysaccharides. However, in such an acid solution, glycoside bonds are always easy to break. This was considered a contributory factor to the extraction rate rapidly increasing with pH levels between 5 and 6. On the contrary, alkaline conditions with a pH higher than 7.0 are conducive to protein extraction. Considering that the purpose of this process is to extract polysaccharides from the *G. candidum* LG-8 cell wall and improve the extraction efficiency of polysaccharides, while reducing the damage to polysaccharide structures and removing the subsequent protein as far as possible, we extracted polysaccharides at pH 7.0, which was one of the optimum conditions in the study.

#### 3.1.4 The influence of intensity

Figure 1D shows the effects of ultrasonic intensity on the release of polysaccharides and proteins. As the ultrasonic intensity increased from 10 W/cm^2^ to 40 W/cm^2^, the extraction efficiency of polysaccharides increased to 18.13%, after which it reached equilibrium. However, the extraction efficiency of proteins reached the highest percentage (5.85%) at 40 W/cm^2^ and then decreased. This indicated that high ultrasonic intensity destroyed the structure of the proteins, eventually resulting in a decrease in released proteins. Therefore, 40 W/cm^2^ was used as one of the optimum conditions to extract polysaccharides in the study. The findings were different from the report by Zhang et al. (20) that showed that the amount of released polysaccharides and proteins increased as ultrasonic intensity increased. On the other hand, Wang et al. (21) reported that the amount of released polysaccharides first increased and then decreased with the increase in ultrasonic intensity. Therefore, the relationship between EPS release in cell walls and ultrasonic intensity is dependent on the strain species and intensity.

#### 3.1.5 The influence of NaCl solutions

A challenge to releasing molecules from microbiological organisms is that these substances are generally embedded in organisms. The extraction is thereby affected by solvents, which were correlated with acoustic cavitation contributing to the extraction efficiency. Therefore, the influence of NaCl solutions on the release of polysaccharides and proteins was studied.

In Figure 1E, the polysaccharide extraction reached equilibrium with a 1.5 M NaCl solution. The extraction rate of proteins reached the maximum value at a 1.0 M NaCl concentration and then decreased. However, when the NaCl solution concentration increased to 0.75 M, the extraction efficiencies of polysaccharides and proteins were 11.74% and 2.31%, respectively. The results showed that the extraction efficiency of proteins was lower than that of polysaccharides. In addition, the extraction efficiency of polysaccharides increased slowly at only 0.64% as the salt concentration increased from 0.75 to 1.5 M. Therefore, to improve the extraction efficiency of polysaccharides from the *G. candidum* LG-8 cell wall, NaCl at a concentration of 0.75 M was selected as the optimum ultrasonic solution. The findings indicated that protein extraction was driven by low levels of NaCl solution but inhibited by high concentrations of NaCl solution. Shih et al. (22) proved that inorganic salts could salt out the protein, which resulted in a decrease in the content of soluble protein. The level of ions changed at high concentrations of salt solution, causing protein molecules to precipitate. The affinity between salt and water molecules is stronger than that between protein and water, resulting in the hydration layer around protein reducing or even disappearing, which makes it difficult to extract protein and also reduces the extracted-soluble protein content.

### 3.2 ABTS radical scavenging ability

Polysaccharides and proteins extracted from microbiological organisms are generally expected to perform certain functions, such as antioxidant activity. In addition, the microbes are a direct source of function, which may be subject to various factors. The study investigated the ABTS radical scavenging capability of *G. candidum* LG-8 cells, focusing on comparisons before and after undergoing ultrasound treatment, as well as examining the antioxidant activity of polysaccharides extracted via ultrasonication.

**Figure 2:**
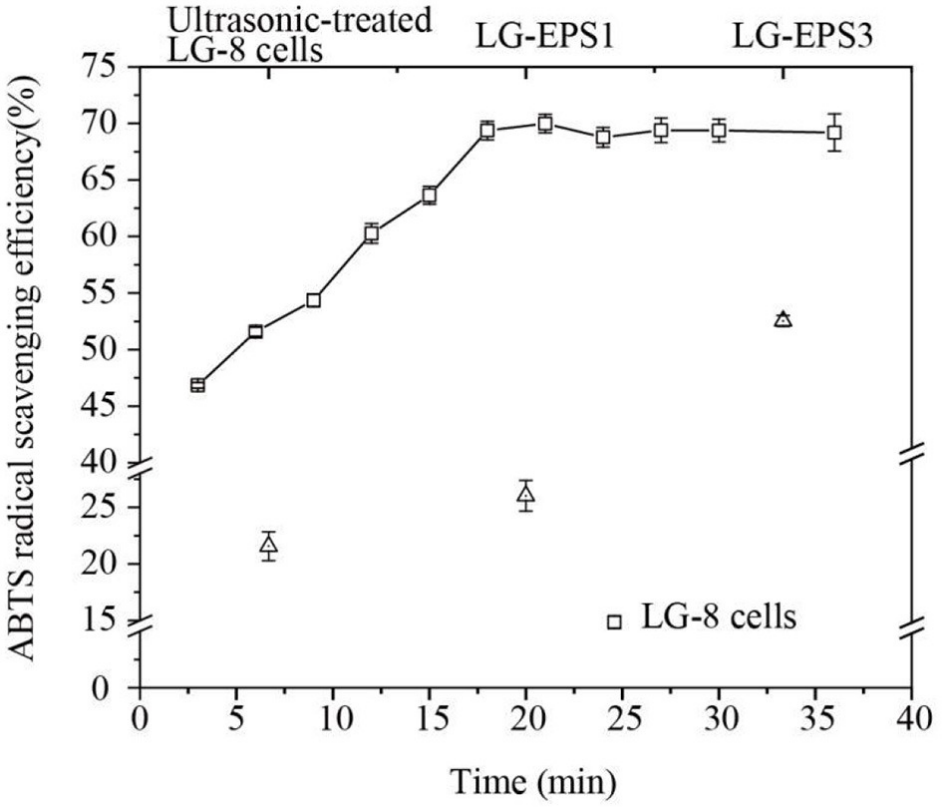
ABTS radical scavenging efficiency of *G. candidum* LG-8 cells, ultrasonic-treated *G. candidum* LG-8 cells, LG-EPS1, and LG-EPS3.

Figure 2 shows that *G. candidum* LG-8 cells (the control group) scavenged 22% of ABTS radicals. However, the cells treated by ultrasound for 3 min and 18 min could remove 46.83% and 69.37% of ABTS radicals, respectively. The significant improvement in scavenging ability suggested that ultrasonic-treatment contributed to the release of antioxidant polymers from fungal cells. Moreover, the ABTS radical scavenging ability of *G. candidum* LG-8 cells was not significantly increased after ultrasonic preparation for 18 min, which was consistent with the changing trend in the extraction efficiency of polysaccharides. Consequently, it is speculated that the released polysaccharide played a major role in scavenging ABTS radicals. This result was consistent with a previous report that showed that antioxidant activities were correlated with the polysaccharide (23, 24). These results will improve understanding of the mechanism of scavenging ABTS radical by LG-8 cells. In addition, to obtain a better antioxidant effect, the extraction efficiency of polysaccharides should be the primary factor considered.

The antioxidant efficiencies of crude (LG-EPS1) and purified polysaccharides (LG-EPS3) at 1 mg/mL were 26.63% and 51.68%, respectively. This indicated that the purification process was beneficial for scavenging ABTS radicals. Small molecules and some proteins were removed from the solvent extracts. Zeng et al. (25) reported that deproteinization led to glycoside bond losses at different degrees (1.14–64.05%) and influenced the antioxidant activity of *Ganoderma lucidum* polysaccharides. Therefore, it is rational that the increase in the antioxidant ability of LG-EPS3 was derived from the purified polysaccharides. Xiao et al. (26) reported that water, methanolic extract, and ethanolic extract of chickpeas at 1 mg/mL were ~20%, 30%, and 25%, respectively. Wang et al. (27) showed that GCP-WS extracted from the fermentation of wheat straw was ~25%. The antioxidant abilities of ultrasound-extracted EPS from *G. candidum* LG-8 were 1.0–2.0-fold of the above mentioned extracts. Lin, Nuerxiati, and Gu et al. (28) showed that the ABTS radical scavenging efficiencies of ultrasound extracts were lower than 50% (10, 29). The above mentioned results, as detailed in Table 1, reveal that ultrasound-treated *G. candidum* LG-8 and its ultrasound extracts had obvious advantages over other materials treated by ultrasound and other methods, which had superiority in scavenging ABTS radicals.

**Table 1:** ABTS radical scavenging efficiency of other extracts from several materials.

**Materials**	**Extracts**	**Concentration (mg/mL)**	**Scavenging activity**	**References**
Chickpeas	Water extracts 80% methanolic extracts, 80% ethanolic extracts	1	~20% ~30%	(26)
Wheat straw	GCP-WS extracted from fermentation broth	1	~25%	(21)
*Ziziphus jujuba* Mill var. spinosa seeds	Ultrasound extracts	1	~5%	(29)
*Orchis chusua* D. Don (Salep)	Ultrasonic-extracted polysaccharides (USP-N and USP-U)	1	~32% and 20%, respectively	(28)
Sagittaria sagittifolia L.	Ultrasonic-extracted polysaccharides (SPU60-W)	1	~48%	(10)

### 3.3 Monosaccharide analysis of LG-EPS1 and LG-EPS3

**Figure 3:**
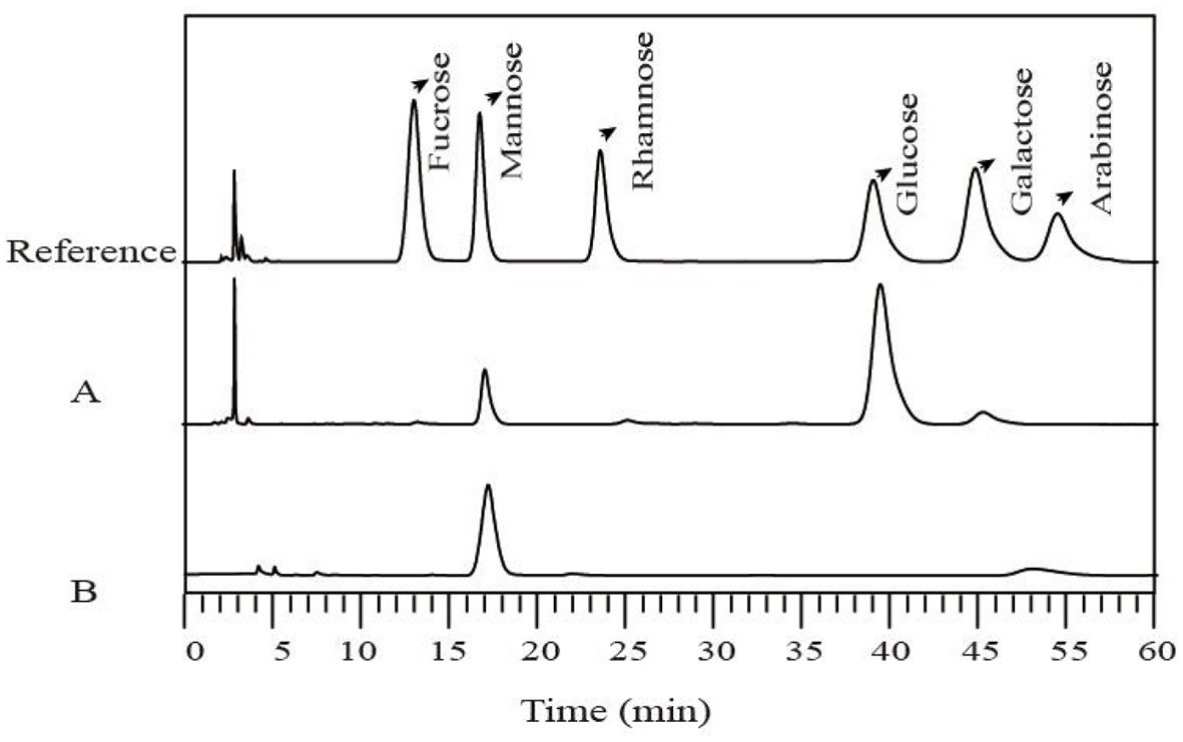
Monosaccharide compositions of LG-EPS1 **(A)** and LG-EPS3 **(B)** measured by HPLC.

The monosaccharide compositions of LG-EPS1 and LG-EPS3 were determined by HPLC which indicated that LG-EPS1 (Figure 3A) was a heteropolysaccharide composed of mannose (18.09%), glucose (77.88%), and galactose (4.03%). However, the column-purified LG-EPS3 (Figure 3B) mainly contained mannose. LG-EPS3 showed higher ABTS radical scavenging capacity than crude LG-EPS1, indicating that a single monosaccharide increased the antioxidant activity. This suggested that the antioxidant activity of polysaccharides is highly correlative to the monosaccharide composition. These above-combined analyses helped us to speculate that monosaccharides are tightly associated with antioxidant activity, which has been proven by a number of previous studies (20, 21). Ni et al. (30) showed that the first canonical correlative coefficient between monosaccharides and the antioxidant activity of polysaccharides is 0.8444. Meng et al. (31) reported that there were high correlations between the antioxidant activities and monosaccharide composition, especially glucuronic acid and glucose content.

### 3.4 TEM and SEM analyses

**Figure 4:**
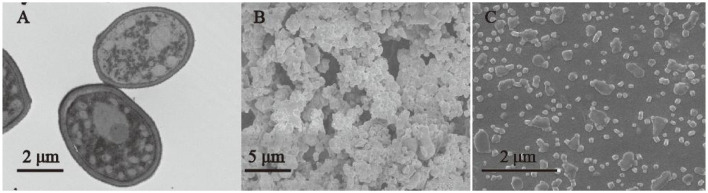
TEM micrograph of *G. candidum* LG-8 cells **(A)** (5 K ×). SEM micrographs of EPS **(B)** LG-EPS1, mag = 5 K ×; **(C)** (LG-EPS3, 50 K ×).

A TEM micrograph (Figure 4A) showed that the *G. candidum* LG-8 cell wall consists of three layers that together are ~300-nm thick. The outside-in layers were reported to be mannan, β-glucan, and chitin (8). It was thereby rational that the high extracting yield of *G. candidum* LG-8 polysaccharides was in part due to the thick cell wall abundant in polysaccharides. Figure 4B showed that the morphology of LG-EPS1 consists of nanospheres with diameters <500 nm. This was different from the typical structure of microbial polysaccharides, which have a porous network and rough surface with detrital and branched structures (23, 32). The microstructure was presented to correlate with physical properties, such as water-holding capacity, determining polysaccharide application in foods, medicine, and environmental protection. Wang et al. (32) showed that it was a potential candidate for the manufacture of plasticized films due to the typical and compact structure of the polysaccharide. Although the morphologies of LG-EPS1 and LG-EPS3 were similar, LG-EPS1 gathered as irregular clumps, whereas LG-EPS3 dispersed more uniformly. Yuan et al. reported that crude polysaccharide exhibited lower apparent viscosities and a smaller particle size than that degraded by ultrasonic treatment (33). However, LG-EPS1 and LG-EPS3 were extracted using the same ultrasonic conditions. Therefore, the presence of scattered nanoparticles was primarily due to the absence of proteins caused by the purification process. Consequently, it was rational that the concentrated polysaccharide with protein removal was correlated with high antioxidant activity in Section 3.2.

Nanotechnology has been applied in several industries, including food, medicine, energy, and agriculture. Natural polysaccharides and their derivatives are widely used to produce nanomaterials. Yang et al. (34) used neutral polysaccharides to fabricate biopolymer complexes. Li et al. (35) used exopolysaccharide nanoparticles for bioremediation. However, natural spherical nanopolysaccharides are rare. Figure 4 shows that LG-EPS1 and LG-EPS3 are nanoparticles with highly efficient antioxidant activity; therefore, they are candidates for use in industry.

### 3.5 FTIR analyses of LG-EPS1 and LG-EPS3

**Figure 5:**
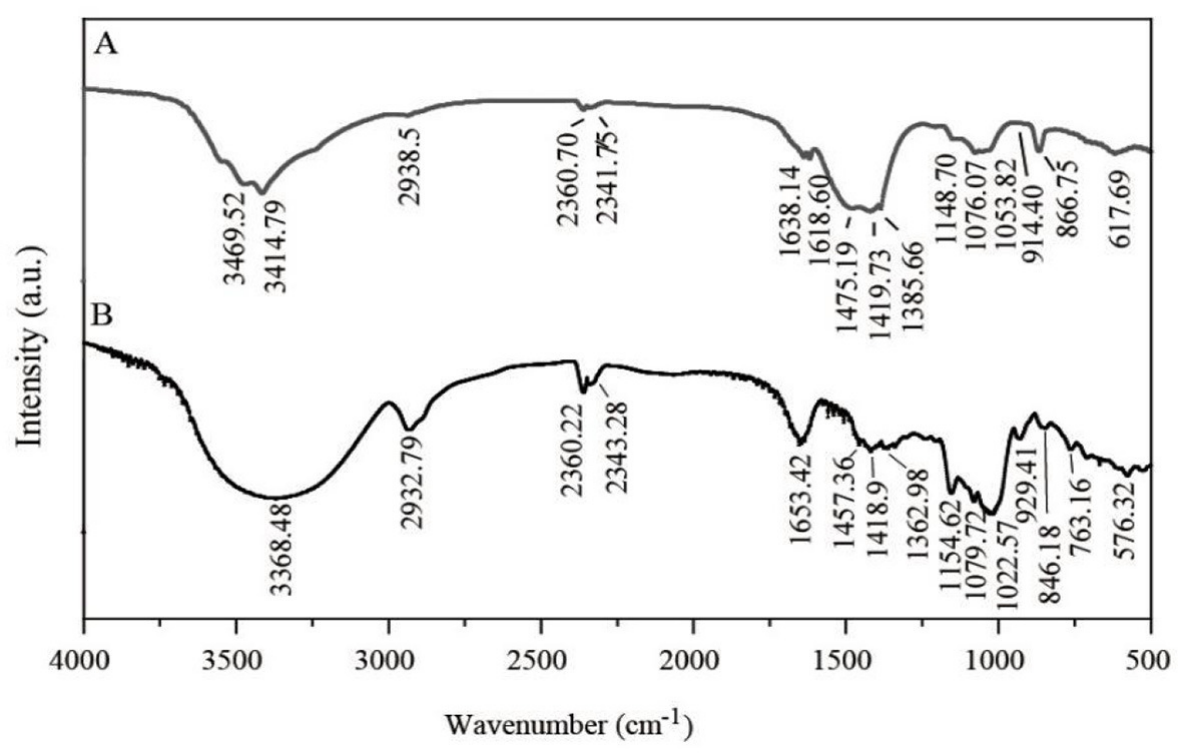
FTIR spectra of LG-EPS1 **(A)** and LG-EPS3 **(B)**. a.u., arbitrary units.

FTIR was applied to identify groups of LG-EPS1 (Figure 5A) and LG-EPS3 (Figure 5B). The peak of the -OH groups was extremely broad in wave numbers ranging from 3500 cm^−1^ to 3200 cm^−1^, and the stretching of -OH groups is associated not only with the production of carboxylic acids but also the adsorption of -CH groups (2968 cm^−1^-2964 cm^−1^) (36, 37). Therefore, the presence of peaks at 3500–2900 cm^−1^ indicated that LG-EPS1 (3469.52 cm^−1^, 3414.79 cm^−1^, and 2938.5 cm^−1^) and LG-EPS3 (3368.48 cm^−1^ and 2932.79 cm^−1^) contain O-H, N-H, and C-H. The characteristic peak at 1652.50 cm^−1^ is a hydration vibration peak of C=O in -NHCOCH3, indicating content of the carbonyl group of a carboxylic acid group in LG-EPS1 (1638.14 cm^−1^) and LG-EPS3 (1653.42 cm^−1^) (36, 37). Moreover, peaks located at 1419.89 and 1489.09 cm^−1^ were assigned to the -COOH group. Vibration of C-O-C, C-O-H link bonds and the hydroxyl of the pyranose ring were reported to be assigned at wavenumbers ranging from 1000 cm^−1^ to 1200 cm^−1^ (36, 37). Angular peaks at wavenumbers 1053.82 cm^−1^ (LG-EPS1) and 1022.57 cm^−1^ (LG-EPS3) thereby indicated the presence of C-O polysaccharides in LG-EPS1 and LG-EPS3 (36, 37). In addition, the characteristic adsorption bands correlated with polysaccharides were observed at wavenumbers 866.75 cm^−1^-914.40 cm^−1^ (LG-EPS1) and 846.18 cm^−1^-929.41 cm^−1^ (LG-EPS3), respectively (38, 39).

## 4 Conclusions

Above all, a water-soluble polysaccharide from yeast-like *Geotrichum* was extracted by ultrasound, and the extraction conditions were optimized. A single factor test showed that the ultrasonic extraction efficiency of polysaccharide reached 18.13%. The ultrasonic-extracted polysaccharide showed remarkable antioxidant ability in scavenging ABTS radicals. In addition, ultrasonic treatment improved the ABTS radical scavenging ability of *G. candidum* LG-8 cells. The results showed that LG-EPS1 was mainly composed of mannose and glucose, in the shape of small spheres gathering as irregular clumps. FTIR results also showed the presence of polysaccharides in the extracts. LG-EPS3 purified by gel chromatography displayed as nanoparticle. Ultrasound was not only applicable in extracting polysaccharides but was also beneficial for improving the scavenging ABTS radical ability of *G. candidum* LG-8 cells. Therefore, the polysaccharides of *G. candidum* LG-8 cell walls are potential antioxidants.

## Data availability statement

The original contributions presented in the study are included in the article/supplementary material, further inquiries can be directed to the corresponding author.

## Author contributions

FJ: Investigation, Visualization, Writing – original draft. XC: Investigation, Visualization, Writing – original draft. XG: Investigation, Writing – original draft. GJ: Resources, Software, Writing – original draft. JW: Resources, Validation, Writing – original draft. LM: Conceptualization, Data curation, Formal analysis, Funding acquisition, Methodology, Project administration, Supervision, Visualization, Writing – review & editing.
